# Horizontally Aligned Carbon Nanotube Based Biosensors for Protein Detection

**DOI:** 10.3390/bioengineering3040023

**Published:** 2016-09-29

**Authors:** Hu Chen, Jingfeng Huang, Derrick Wen Hui Fam, Alfred Iing Yoong Tok

**Affiliations:** 1 School of Materials Science and Engineering, Nanyang Technological University, Singapore 639798, Singapore; chen0805@ntu.edu.sg (H.C.); huang_jingfeng@rp.edu.sg (J.H.); 2 Institute for Sports Research, Nanyang Technological University, Singapore 639798, Singapore; 3 Department of Chemistry, Loughborough University, Loughborough LE11 3TU, UK; 4 School of Engineering, Republic Polytechnic, Singapore 738984, Singapore; 5 Department of Chemistry, Royal College of Science, Imperial College, London SW7 2AZ, UK; derrickfamwh@imre.a-star.edu.sg

**Keywords:** horizontal alignment, CNT-based biosensor, protein detection

## Abstract

A novel horizontally aligned single-walled carbon nanotube (CNT) Field Effect Transistor (FET)-based biosensing platform for real-time and sensitive protein detections is proposed. Aligned nanotubes were synthesized on quartz substrate using catalyst contact stamping, surface-guided morphological growth and chemical vapor deposition gas-guided growth methods. Real-time detection of prostate-specific antigen (PSA) using as-prepared FET biosensors was demonstrated. The kinetic measurements of the biosensor revealed that the drain current (I_d_) decreased exponentially as the concentration of PSA increased, indicating that the proposed FET sensor is capable of quantitative protein detection within a detection window of up to 1 µM. The limit of detection (LOD) achieved by the proposed platform was demonstrated to be 84 pM, which is lower than the clinically relevant level (133 pM) of PSA in blood. Additionally, the reported aligned CNT biosensor is a uniform sensing platform that could be extended to real-time detections of various biomarkers.

## 1. Introduction

Since its discovery in 1991 by Iijima [1], carbon nanotubes (CNT) have attracted increasing attention owing to their extraordinary mechanical properties [2,3], high thermal conductivity [4], large aspect ratio [5], and high electron mobility [6]. CNTs can be categorized as single-walled carbon nanotubes (SWCNT) and multi-walled carbon nanotubes (MWCNT). SWCNT consist of a single atomic layer of a carbon cylinder with an outer diameter ranging from 10 to 30 Å and MWCNT consist of multiple single atomic layers of carbon cylinders within each other, with the outermost diameter ranging between 20 and 200 Å [7]. Ijima et al. first reported the observation of MWCNT using the arc-discharge technique [1,8]. Based on that, Bethune reported the synthesis of SWCNT using the arc-discharge method and a metal catalyst [9].

Currently, three techniques have been widely used for CNT growth: the carbon arc-discharge technique [9,10], the laser ablation technique [11,12] and chemical vapor deposition (CVD) [13,14,15]. Among these approaches, CVD is the most commonly used. In this process, a carrier gas (e.g., nitrogen) and a carbon-contained gas (e.g., ethanol) are mixed in the reactor and contact with the metal catalysts deposited on the substrate surface. As a result, the growth of nanotubes is initiated on the surface of the catalyst particles. It has been demonstrated that the type of CNT synthesized is dependent on the reaction temperature and catalyst particle size [16], although the exact mechanism needs further clarification. Also, the diameter of nanotubes obtained and the average growth rate increases as the chamber pressure decreases. Nevertheless, this one-dimensional material has anisotropic characteristics, meaning that the mechanical, optical, electronic properties along the tube axis are significantly different from those along the perpendicular axis [17]. As a result, the sensing performance of conventional CNT-based platforms (e.g., field-effect transistors) [18], which rely on a dense network of randomly arranged CNT [19], is severely limited. Therefore, efforts have been made to enhance various properties of CNT by involving horizontal alignment. Thess et al. reported the synthesis of aligned SWCNT using the laser ablation technique [11]. To date, approaches reported for the growth of horizontally aligned CNT include in situ growth using electron cyclotron resonance CVD [20] or atmospheric pressure CVD [21] and post growth using Langmuir-Blodgett assembly [22], spin-coating [23], etc. Indeed, it has been demonstrated that devices based on horizontally aligned CNTs exhibit various advantages over those based on randomly distributed CNT, including reduced tube-to-tube resistance [24,25] and minimized electrostatic screening [26,27,28]. For SWCNT growth by CVD, morphology control can be achieved using surface control techniques. Conventionally, sapphire was used to provide step-edge interactions between the growing SWCNT and the substrate surface. In this case, quartz was used as the substrate to enhance the density and horizontal alignment of SWCNT produced [29,30]. However, misalignment issues still persist and may have a significant effect on the transport properties of the SWCNT due to bridging effects, despite their minor extent. Additionally, it has been demonstrated that the degree of misalignment can be exacerbated as the density of nanotubes increases. Therefore, it is of great importance to optimize the synthesis parameters, including the annealing temperature, catalyst concentration, precursor flow rates and growth temperature, to further improve the alignment of SWCNT [31,32].

Prostate-specific antigen (PSA, the protein structure shown in Figure S1 in the Supplementary Materials) is a glycoprotein enzyme that is generated for dissolving the ejaculate and cervical mucus [33]. Present in small quantities in the serum of male individuals with healthy prostates, this enzyme is often elevated in the presence of prostate cancer or other prostate disorders [34]. Recently, detection of PSA has attracted increasing attention and various studies in this field have been reported. Among all the detection methods, ELISA (enzyme-linked immunosorbent assay) is the most widely used for PSA detection and its sensitivity can be higher than 4 ng/mL (equivalently 133 pM) [35,36], which is the reported critical level of PSA in blood [37]. Nevertheless, ELISA is a resource-intensive assay that requires sophisticated instruments and well-trained personnel [38].

**Figure 1:**
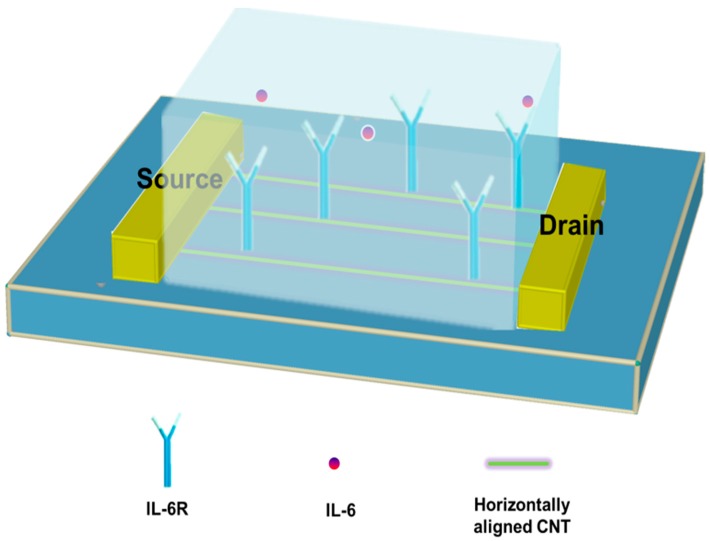
Schematic illustration of liquid-gated field effect transistor (FET) sensors based on horizontally aligned carbon nanotubes for detection of prostate-specific antigen.

In this article, we report a facile method for the synthesis of horizontally aligned CNTs on quartz substrate and demonstrate detection of prostate specific antigen (PSA) using a sensitive real-time biosensing platform based on the as-prepared CNT samples. Parameters in the process of CNT growth by CVD were optimized based on experimental results. A schematic illustration of the liquid-gated sensor based on horizontally aligned CNT for PSA detection is shown in Figure 1.

## 2. Experimental

### 2.1. Materials

The quartz wafers purchased from Hoffman Materials Inc. (Kemptville, ON, Canada) were annealed at 900 °C for 8 h and cut into 1 cm × 1 cm slices prior to CVD growth. PSA and its antibody were purchased from United States Biological (Salem, MA, USA). Siloxane (Sylgard 184) and silicon adhesive (3140 RTV Coating) were purchased from Dow Corning, Singapore. The catalyst Co(C_2_H_3_O_2_)_2_·4H_2_O (Cobalt(II) acetate tetrahydrate, ACS reagent, ≥98%) and other reagents for CNT growth were purchased from Sigma-Aldrich Inc. (Singapore) and used without further purification.

### 2.2. Methods

**Figure 2:**
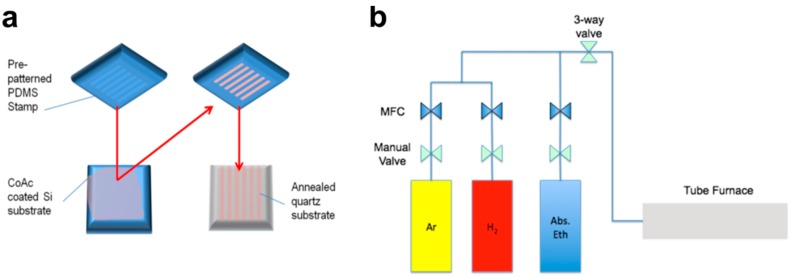
(**a**) Deposition of catalyst on quartz substrate using a polydimethylsiloxane stamp; (**b**) Schematic illustration of the chemical vapor deposition setup used for carbon nanotube growth.

*Sample preparation:* The quartz substrates were firstly ultrasonicated in acetone, isopropyl alcohol and deionized water for 5 min each to remove surface contaminants. The substrates were then annealed with a 4 h ramp from room temperature, held at 850 °C for 8 h before cooling down to room temperature in 4 h. Polydimethylsiloxane (PDMS) stamps, which were fabricated by curing siloxane over a silicon mask with the desired pattern (10 μm stripes with a spacing of 100 μm), were utilized for micro-contact printing of catalyst on quartz substrates. The Cobalt(II) acetate tetrahydrate catalyst was dissolved in absolute ethanol solution at a concentration of 0.4 mg/ml and applied on the quartz substrate via micro-contact printing using the as-prepared PDMS stamp. More specifically, the catalyst was first deposited onto a flat piece of Si, and then deposited on the quartz surface by bringing the stamp into conformal contact with the substrate for 2 min, as illustrated in Figure 2a. Finally, the stamp was removed and the catalyst-deposited substrate was dried naturally.

*CNT growth by chemical vapor deposition*: Subsequently, horizontally aligned SWCNTs were grown on the annealed quartz substrate using CVD method with ethanol as the carbon source [39]. As shown in Figure 2b, the CVD setup used for CNT growth consists of two gas containers with a carrier gas (Ar) and a reducing gas (H_2_) respectively, a feedstock (absolute ethanol in this case) container and a process chamber that can heat up to 1000 °C. The flow of the gases was controlled by mass flow controllers (MFCs) at each individual line. The catalyst-deposited substrates were annealed in air at 850 °C for 5 min to allow catalyst oxidation. Then, catalyst reduction was performed at a constant flow of Ar/H_2_ (ratio = 100 sccm:150 sccm) at 850 °C for another 5 min to obtain Co nanoparticles for CNT growth in the next stage. Finally, the chamber was heated up to 950 °C and absolute ethanol was introduced by a flow of Ar/H_2_ (ratio = 75 sccm:35 sccm).

*Sample characterization:* The as-prepared samples were examined using field-effect scanning electron microscopy (FESEM, JSM 6340F, JEOL, Tokyo, Japan) and atomic force microscopy (AFM, Dimension 3100, Plainview, NY, United States) to investigate the horizontal alignment and density of carbon nanotubes grown on quartz substrates.

*Fabrication of liquid gated FET sensors*: 2-mm-wide, 100-nm-thick Au source and drain electrodes (spacing = 200 µm) were evaporated onto the substrates were evaporated on the substrates, followed by removal of nanotubes outside the electrodes region to avoid undesired bridging. Finally, a silicone rubber reservoir was established using the silicon adhesive to confine the test solution. Figure S2 (shown in Supplementary Materials) shows a typical device fabricated using the proposed methodology. The I–V curves of the transistors fabricated were obtained using a methodology similar to a previous report [40]. With a small voltage bias (V_d_) of 10 mV applied between the source electrode and the drain electrode, the current (I_d_) flowing through the SWCNTs was recorded while sweeping the gate voltage (V_g_) from −600 mV to 0 mV. I_d_–V_d_ plots at different gate voltages were also recorded for electrical characterization.

*Kinetic measurements:* Antibodies were firstly immobilized on carbon nanotubes using 1-pyrenebutanoic acid succinimidyl ester (PBSE, chemical structure shown in Figure S3 in the Supplementary Materials) as the linker [41]. Briefly, the device was incubated with PBSE (5 mM in phosphate buffered saline buffer, pH = 7.4) for 1 h, rinsed with PBS (phosphate buffer saline) for several times to remove loosely bound antibodies and then incubated with PSA antibody for 1 h. A voltage bias of 10 mV was applied between the source electrode and the drain electrode, while the gate potential was applied via a reference electrode (3 M KCl) (FLEXREF from World Precision Instruments). Subsequently, devices were incubated with solutions with different concentrations of target protein (500 pM, 1 nM, 10 nM, 100 nM and 1 μM) for 15 min each. After protein incubation, the device was rinsed for several times and the drain current was measured in PBS.

## 3. Results and Discussion

**Figure 3:**
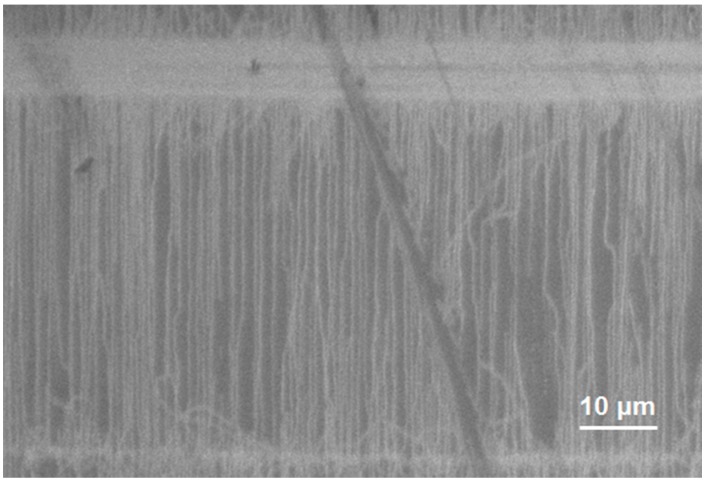
Field emission scanning electron microscopy image of horizontally aligned carbon nanotubes on quartz substrate.

**Figure 4:**
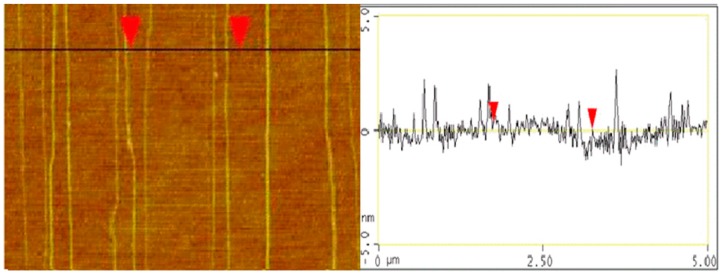
Atomic force microscopy image and the corresponding section analysis of horizontally aligned carbon nanotubes on quartz substrate.

**Figure 5:**
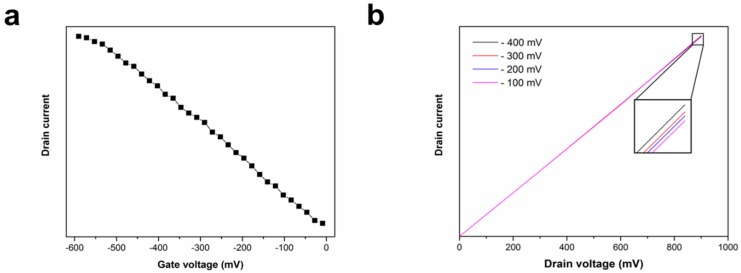
Atomic force microscopy image and the corresponding section analysis of horizontally aligned carbon nanotubes on quartz substrate.

Figure 3 shows the FESEM image of SWCNT on quartz substrates. As observed, SWCNT with excellent horizontal alignment have been grown on quartz substrates. Figure 4 shows the AFM image and the corresponding section analysis of SWCNT grown on quartz substrate. As can be seen, the average diameter of SWCNT grown on quartz substrate was 2 nm, which is consistent with previous reports [42,43]. Figure 5a,b show the I_d_ vs. V_g_ at a drain voltage of 10 mV and I_d_ vs. V_d_ at different gate voltages (−400 mV, −300 mV, −200 mV and −100 mV), respectively. As observed from Figure 5a, the device shows a typical semiconducting behavior in the range of −600 mV to 0 mV, indicating good functionality as a transistor [44]. Additionally, the linear relation between I_d_ and V_d_ illustrates Ohmic contacts between the carbon nanotubes and the Au electrodes.

**Figure 6:**
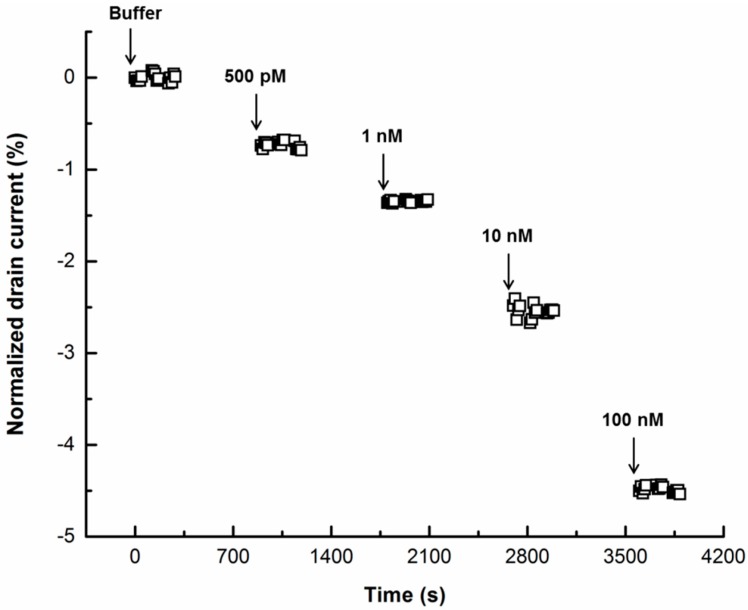
Kinetic measurements of prostate-specific antigen at different concentrations using as-prepared devices at a fixed gate voltage (−300 mV).

Before kinetic measurements, antibody-immobilized devices were incubated with PBS and the drain current (I_d_) was allowed to stabilize so that the effects of non-specific binding of contaminants in buffer are excluded; the value of I_d_ observed at this stage was regarded as the baseline for successive measurements. Then, the device was incubated with a solution with different concentrations of PSA (500 pM, 1 nM, 10 nM, 100 nM and 1 μM) for 15 min each, followed by buffer rinsing. I_d_ was monitored after the 15 min incubation at a fixed gate voltage of −300 mV and the results are shown in Figure 6. In essence, significant I_d_ reductions were observed in the presence of PSA and the reductions were proportional to the PSA concentration. This can be attributed to the positive surface charge potential of PSA molecules [45], which has an equivalent effect to increased negative gate voltage, thus shifting the conductance curve upon binding to the antibodies on the sensor surface. On the other hand, the electrostatic interaction between adsorbed molecules and SWCNTs caused by adsorptions of positively charged molecules also contributes to reductions in I_d_ [46]. The limit of detection (LOD) achieved, calculated using the 3σ/S approach [47,48], was 84 pM. More specifically, the LOD was calculated using the following equation:

(1) $$\mathrm{LOD}=\frac{3\sigma }{S}$$

where σ is the standard deviation of device responses to PBS buffer and S is the sensitivity, which is defined as the slope of the linear sensor response range and can be calculated using the following equation:

(2) $$S=\frac{{I_{d}}^{\prime }-I_{d}}{C^{\prime }-0}$$

Based on the results of the kinetic measurements obtained, it can be concluded that the detection of PSA at clinically relevant levels (133 pM in serum) can be achieved by the proposed horizontally aligned CNT-based biosensor.

**Figure 7:**
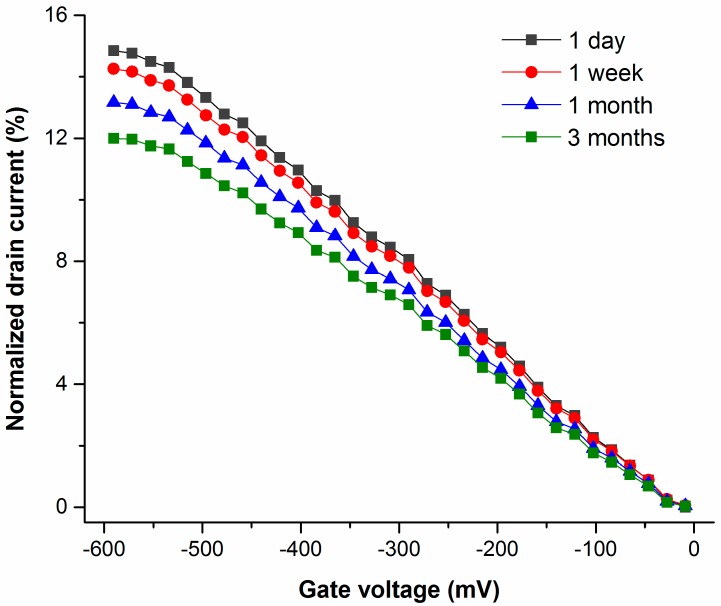
I_d_ vs. V_g_ of devices after storage under ambient conditions for different durations.

Additionally, the stability of devices proposed in ambient conditions was investigated. As shown in Figure 7, ∆ I_d_ in response to the same variation of V_g_ (−600 mV to 0 mV) were 15%, 14.2%, 13% and 12% for devices after storage for one day, one week, one month and three months, respectively. The degradation of sensitivity (in terms of I_d_ vs. V_g_) of the device developed dropped by approximately 20% after storage under ambient conditions for three months, indicating excellent device stability (there was no significant performance degradation after storage under ambient conditions for up to three months).

## 4. Conclusions

A facile method for the synthesis of horizontally aligned CNTs on quartz substrate and their applications for protein detection have been proposed. Excellent horizontal alignment has been achieved by optimizing parameters in the process of CNT growth by CVD. Also, detection of PSA using a sensitive real-time biosensing platform based on the as-prepared CNT samples has been demonstrated and the LOD achieved was 84 pM, indicating that the fabricated sensor can be used for the detection of PSA at clinically relevant levels. Additionally, this universal platform exhibits a great potential for clinical applications as it can be easily extended to the detection of various targets upon functionalization with the corresponding receptors.

## Supplementary Materials

The following are available online at http://www.mdpi.com/2306-5354/3/4/23/s1, Figure S1: protein structure of prostate specific antigen, Figure S2: an field-effect transistor sensor developed by the method described. The silicon rubber reservoir is to confine the solutions and the silver paint is coated for the convenience of probing, Figure S3: chemical structure of 1-pyrenebutanoic acid succinimidyl ester (PBSE). The pyrenebutanoic group adheres to carbon nanotubes via π–π interaction, while the succinimidyl group reacts with amine group on protein to form covalent bonds.
