# Supplementary material for: Horizontally Aligned Carbon Nanotube Based Biosensors for Protein Detection
Source: Bioengineering (Basel). 2016 Sep 29;3(4):23. doi: 10.3390/bioengineering3040023 (PMC5597266; doi:10.3390/bioengineering3040023)
Supplement: Supplementary file 1 [file bioengineering-03-00023-s001.pdf]

bioengineering

Supplementary Materials: Horizontally Aligned Carbon Nanotube Based Biosensors for Protein Detection

Hu Chen, Jingfeng Huang Derrick, Wen Hui Fam and Alfred Iing Yoong Tok

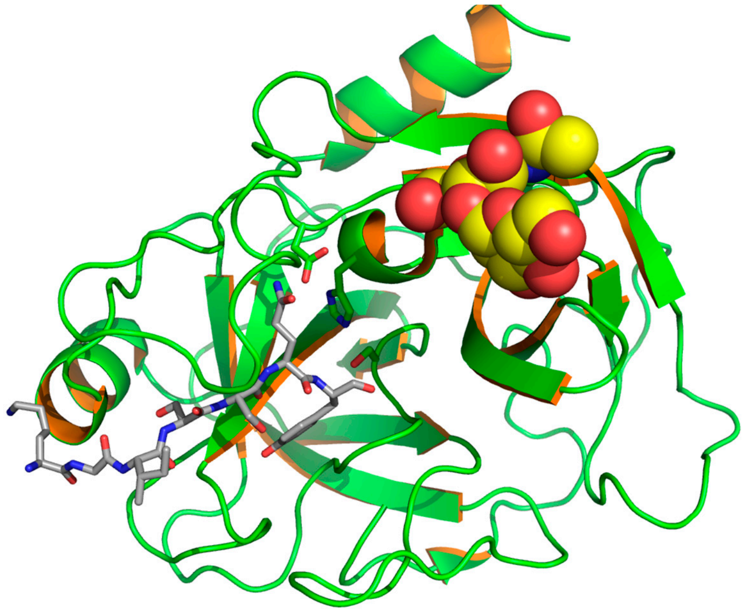

Figure S1. Protein structure of prostate specific antigen.

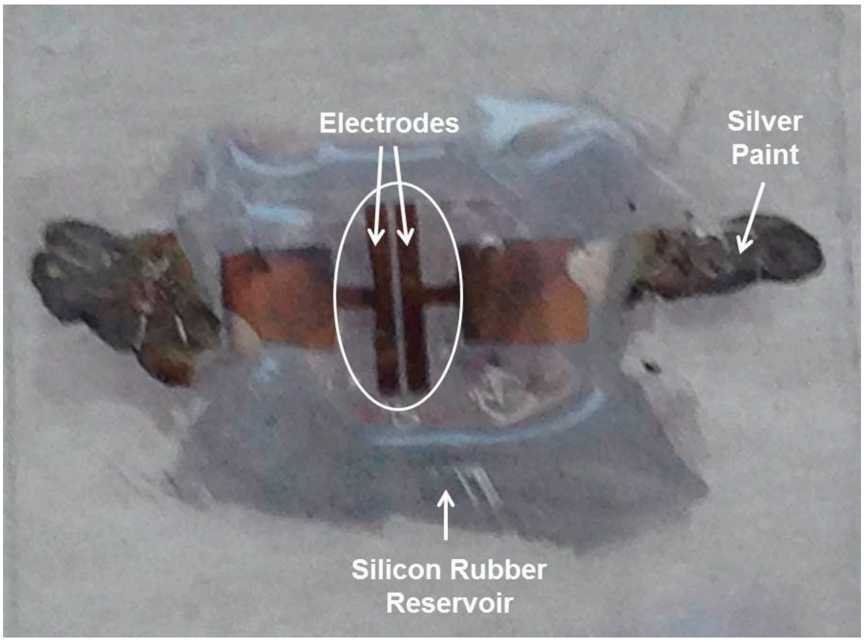

Figure S2. An field-effect transistor sensor developed by the method described. The silicon rubber reservoir is to confine the solutions and the silver paint is coated for the convenience of probing.

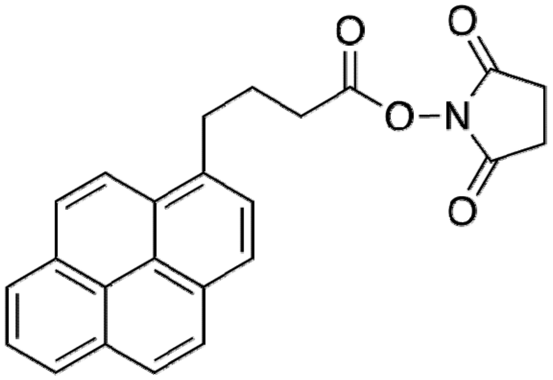

Figure S3. Chemical structure of 1-pyrenebutanoic acid succinimidyl ester (PBSE). The pyrenebutanoic group adheres to carbon nanotubes via π–π interaction, while the succinimidyl group reacts with amine group on protein to form covalent bonds.

© 2016 by the authors; licensee MDPI, Basel, Switzerland. This article is an open access article distributed under the terms and conditions of the Creative Commons by Attribution (CC-BY) license (http://creativecommons.org/licenses/by/4.0/).
